# Rechargeable calcium phosphate orthodontic cement with sustained ion release and re-release

**DOI:** 10.1038/srep36476

**Published:** 2016-11-03

**Authors:** Ling Zhang, Michael D. Weir, Laurence C. Chow, Mark A. Reynolds, Hockin H. K. Xu

**Affiliations:** 1 State Key laboratory of Military Stomatology, Department of Prosthodontics, School of Stomatology, Fourth Military Medical University, Xi’an, 710032, China; 2 Department of Endodontics, Prosthodontics and Operative Dentistry, University of Maryland Dental School, Baltimore, MD 21201, USA; 3 Dr. Anthony Volpe Research Center, American Dental Association Foundation (ADAF), National Institute of Standards and Technology, Gaithersburg, MD 20899, USA; 4 Center for Stem Cell Biology & Regenerative Medicine, University of Maryland School of Medicine, Baltimore, MD 21201, USA; 5 Department of Mechanical Engineering, University of Maryland, Baltimore County, MD 21250, USA

## Abstract

White spot lesions (WSL) due to enamel demineralization are major complications for orthodontic treatments. Calcium phosphate (CaP) dental resins with Ca and P ion releases are promising for remineralization. However, previous Ca and P releases lasted for only weeks. Experimental orthodontic cements were developed using pyromellitic glycerol dimethacrylate (PMGDM) and ethoxylated bisphenol A dimethacrylate (EBPADMA) at mass ratio of 1:1 (PE); and PE plus 10% of 2-hydroxyethyl methacrylate (HEMA) and 5% of bisphenol A glycidyl dimethacrylate (BisGMA) (PEHB). Particles of amorphous calcium phosphate (ACP) were incorporated into PE and PEHB at 40% filler level. Specimens were tested for bracket-enamel shear bond strength, water sorption, CaP release, and ion recharge and re-release. PEHB+40ACP had higher bracket-enamel bond strength and ion release and rechargeability than PE+40ACP. ACP incorporation into the novel orthodontic cement did not adversely affect the bracket-enamel bond strength. Ion release and re-release from the novel ACP orthodontic cement indicated favorable release and re-release patterns. The recharged orthodontic cement could release CaP ions continuously for four weeks without further recharge. Novel rechargeable orthodontic cement containing ACP was developed with a high bracket-enamel bond strength and the ability to be repeatedly recharged to maintain long-term high levels of CaP ion releases.

The demineralization of tooth enamel adjacent to orthodontic brackets leads to white spot lesions (WSL), a serious and common complication for orthodontic treatments^1^,^2^,^3^,^4^. It has been reported that WSL could take only 1 month to develop^4^ and the incidence of WSL in patients with fixed orthodontic treatments ranged from 73% to 95%^2^,^5^. The incidence of WSL formation could be attributed to the irregular surfaces of brackets, bands, wires and other attachments that provide areas for bacterial and plaque accumulation^6^. Indeed, the existence of these small and complex pieces of equipment makes tooth brushing and cleaning difficult^7^. Furthermore, they limited the natural self-cleansing actions by saliva, oral musculature and tongue^7^,^8^. These conditions promoted the accumulation of plaque and the colonization of cariogenic bacteria^7^,^8^,^9^, which could produce organic acids to form WSL.

Strategies were investigated to manage WSL by preventing enamel demineralization or promoting remineralization^10^,^11^,^12^. Fluoride was used to prevent caries and remineralize tooth structures. Topical fluoride or fluoride-releasing cements showed positive effects on preventing demineralization of enamel surrounding the orthodontic brackets^10^,^11^. However, fluoride treatments of the enamel prior the placement of orthodontic brackets will result in the enamel being more resistant to the phosphoric acid etching. This could decrease the bond strength and lead to pre-mature bond failure^12^. The home use of topical fluoride is frequently not adequate due to low patient compliance^13^. Another promising approach is based on calcium phosphate (CaP) remineralization^14^,^15^,^16^,^17^. Calcium (Ca) and phosphate (P) ions released from CaP biomaterials can produce a Ca and P ion reservoir in dental plaque and onto the tooth surfaces, which can maintain supersaturating levels of Ca and P ions, thus help prevent demineralization and facilitate remineralization. Amorphous calcium phosphate (ACP), casein phosphopeptide amorphous calcium phosphate (CPP-ACP) and fluoride containing-CPP-ACP were incorporated into methacrylate composites, gums, pastes and other dental products and achieved promising effects on caries prevention and enamel remineralization^14^,^15^,^16^,^17^.

Recently, particles of amorphous calcium phosphate (ACP) were incorporated into dental restorative materials and showed superior effects on both caries inhibition and tooth lesion remineralization^18^,^19^,^20^. ACP-containing bonding agents and composites produced high levels of Ca and P ion release^21^,^22^. The potential of ACP as an acid neutralizing agent by way of increased Ca and P ion release at pH of 4 levels, when caries is most prone to develop could be essential^23^. The incorporation of up to 40% of ACP into a dentin adhesive did not negatively affect the dentin bond strength^21^,^22^,^24^.

Previous CaP-containing resins released Ca and P for weeks to a few months, resulting in diminished Ca and P^25^,^26^,^27^. While fluoride-releasing materials were shown to be rechargeable, no rechargeable CaP resins had been reported. The average orthodontic treatment time ranges 14–33 months^28^; therefore, Ca and P ion releases lasting much longer than weeks are needed. Recently, novel rechargeable ACP-containing dental resins were developed for the first time, using monomers of pyromellitic glycerol dimethacrylate (PMGDM) and ethoxylated bisphenol A dimethacrylate (EBPADMA) in combination with fillers of ACP. The new CaP composite and bonding agent demonstrated excellent Ca and P ion rechargeability, yielding sustained long-term Ca and P ion releases for the first time^29^,^30^,^31^. In the present study, the novel CaP recharge method was applied to the development of a rechargeable CaP-containing dental cement and in particular, for orthodontic applications to inhibit enamel demineralization and WSL formation.

Therefore, the objectives of this study were to develop a rechargeable CaP-containing dental cement and investigate the effects of ACP incorporation on orthodontic bracket-enamel bond strength, water sorption, as well as Ca and P ion recharge and re-release. The following hypotheses were tested: (1) The new rechargeable CaP orthodontic cement would yield a bracket-enamel bond strength similar to commercial orthodontic cements without CaP ion release and recharge; (2) the enamel bond strength and the Ca and P ion recharge and re-release efficacy would depend on the cement resin composition; and (3) the new CaP orthodontic cement would exhibit continuous ion release, and the ion release would not decrease with increasing number of recharge and re-release cycles.

## Materials and Methods

### Preparation of ACP

ACP [Ca_3_(PO_4_)_2_] were synthesized via a Spry-drying technique as previously described^18^,^19^. Briefly, calcium carbonate and dicalcium phosphate were dissolved into an acetic acid solution. The concentrations of Ca and P ions were 8 mmol/L and 5.333 mmol/L, respectively, yielding a Ca/P molar ratio of 1.5. The solution was sprayed into a heated chamber, allowing the evaporation of water and the volatile acid. The dried ACP were collected via an electrostatic precipitator, yielding ACP with a mean particle size of approximately 116 nm^19^.

### Formulation of experimental orthodontic cements

Two experimental orthodontic cement compositions were formulated. The first consisted of pyromellitic glycerol dimethacrylate (PMGDM) (Hampford, Stratford, CT) and ethoxylated bisphenol A dimethacrylate (EBPADMA) (Sigma-Aldrich, St, Louis, MO) at a mass ratio of 1:1. Camphorquinone (CQ) (Irgacure819, Ciba Chemicals, Japan) at 0.2% by mass was added for photo-polymerization. Benzoyl peroxide (BPO) at 0.8% by mass (Irgacure819, Ciba Chemicals, Japan) was added to enable a chemical cure. PMGDM is an acidic adhesive monomer^32^,^33^, which can chelate with calcium ions from the recharge solution to achieve recharge capability^30^,^31^. The PMGDM-EBPADMA group is referred to as PE.

To formulate the second resin, 10% of 2-hydroxyethyl methacrylate (HEMA) (Esstech, Essington, PA) and 5% of bisphenol A glycidyl dimethacrylate (BisGMA) (Esstech) were added to the PMGDM-EBPADMA mixture. HEMA and BisGMA are widely used dental monomers. HEMA is an excellent adhesion-promoting monomer due to its hydrophilicity^33^. The mass fractions were selected because previous studies showed that the addition of 10% HEMA and 5% BisGMA into the PMGDM-EBPADMA resin increased the bond strength to dentin^30^. This second group is denoted PEHB. ACP fillers were added into both resins at mass fractions of 0% and 40%, following previous studies^19^,^20^. ACP filler levels >40% were not used due to a decrease in bracket-enamel bond strength in preliminary study.

Two commercial materials served as comparative controls in orthodontic bracket-enamel bond strength testing. Transbond XT (3M Unitek, Monrovia, CA) has been used as an orthodontic cement. The Transbond XT was used in accordance with the manufacturer’s instructions for use. According to the manufacturer, it consisted of silane-treated quartz (70–80% by weight), bisphenol A diglycidyl ether dimethacrylate (10–20%), bisphenol-A-bis (2-hydroxyethyl) dimethacrylate (5–10%), silane-treated silica (<2%) and diphenyliodonium hexafluorophosphate (<0.2%). Transbond is referred to as TB control. Vitremer (3M ESPE, St Paul, MN, USA) is a restorative material and the indications do not include orthodontic bracket cementation. In this study the material was used as a commercial comparative control. Vitremer was used with the primer and a mixture of powder:liquid mass ratio of 2.5:1. Vitremer consisted of fluoroaluminosilicate glass and a light-sensitive, aqueous polyalkenoic acid. Vitremer is referred as VT control. Therefore, four experimental orthodontic cements and two commercial control cements were tested for bracket-enamel bond strength:

1. PMGDM-EBPADMA (referred to as PE);
2. PE+40% ACP (referred to as PE+40ACP);
3. PMGDM-EBPADMA-HEMA-BisGMA (referred to as PEHB);
4. PEHB+40% ACP (referred to as PEHB+40ACP);
5. TB control;
6. VT control.

### Orthodontic bracket shear bond testing and the adhesive remnant index (ARI)

One hundred and twenty extracted human third molars were collected after informed consent was obtained from the patients. The protocol employed was approved by University of Maryland Baltimore Institutional Review Board. All the experiments were carried out in accordance with the approved guidelines and regulations. The teeth were stored in 0.01% thymol solution at 4 °C and used within 1 month after extraction. The orthodontic bracket shear bond strengths were tested as previously described^34^. Each tooth was embedded vertically in a self-curing acrylic resin (Lang Dental Manufacturing, Wheeling, IL) taking into account the buccal axis of the clinical crown, so that the labial surfaces would be parallel to the force during the shear bond test. The coronal portion was submitted to prophylaxis with oil-free pumice and rubber cups at a low speed for 10 seconds (s). The buccal surface was etched with 35% phosphoric acid (Scotchbond, 3M ESPE, St. Paul, MN) for 30 s, then washed and dried. Each cement was applied to the base of the bracket, which was placed on the center of the tooth surface with a firm pressure. Excessive cement around the bracket was removed. The cement was polymerized from all four sides (mesial, distal, occlusal, and gingival) of the bracket for 10 s each, for a total of 40 s, using a halogen light-curing unit (Optilux VCL 401, Demetron Kerr, Danbury, CT) with a standard mode. The output intensity was monitored to be the same. The light unit was equipped with a standard light guide and the tip of the guide was kept at approximately 1 mm from the bracket. The specimens were stored in distilled water at 37 °C for 24 hours. All the brackets were cemented by an experienced clinician who was blinded to which kind of cement was used during bracket placement. Then the specimens in each group were divided into two subgroups. One subgroup was subjected to shear bond strength testing after the samples were stored in water for 24 hours (referred to as 1 day in water, n = 10). The other subgroup (n = 10) was immersed in a demineralization solution of pH 4 for 1 month. The demineralizing solution consisted of 3.0 mmol/L CaCl_2_, 1.8 mmol/L K_2_HPO_4_, 0.1 mol/L lactic acid, and 1% carboxymethylcellulose, with pH 4 adjusted with the addition of KOH^27^. This mimicked the demineralizing biofilm acids that the orthodontic cement-enamel bonded areas would experience *in vivo* which could potentially degrade the bond strength^27^. For the 1 month aging, each bonded tooth was inversely placed in a closed tube with the bonded interface being completely immersed in 0.5 mL of demineralization solution. The solution was changed daily so that a fresh pH 4 solution was used every day. The pH 4 was used because in the oral environment, acidogenic bacteria ferment carbohydrates and produce organic acids which can decrease the local plaque pH to 4.5 or 4^35^,^36^. The purpose of aging in the demineralizing solution was to produce a cariogenic challenge on the bonded interface in order to evaluate the bond durability under acid challenges^37^. To measure the shear bond strength, a chisel on a Universal Testing Machine (MTS, Eden Prairie, MN) was positioned to the upper part of the bracket base and parallel to the bonded interface. An occlusogingival load was applied at a cross-head speed of 0.5 mm/min until the bracket detached. Orthodontic bracket shear bond strength = load at failure/bracket surface area^38^,^39^.

After brackets were detached, each tooth surface was observed under a stereomicroscope (Leica Zoom 2000, Leica Microsystems GmbH, Wetzlar, Germany) to examine the failure mode. The Adhesive Remnant Index (ARI) was based on the remaining cement material on enamel, using the following criteria^34^: 0 = no cement remained on enamel; 1 = less than half of the cement remained on enamel; 2 = more than half of the cement remained on enamel; 3 = all the cement remained on enamel.

### Water sorption assessment

The six groups of materials were used for water sorption assessment. Each cement paste was placed into a plastic mold with 12 mm diameter and 1.5 mm thickness. The specimen was light-cured (Triad 2000, Dentsply, York, PA) for 1 min on each open side of the mold, and then incubated at 37 °C for 24 h. To measure water sorption, the cement disks were first dried with desiccant (WA Hammond Drierite, Xenia, OH) in a container under vacuum to a constant mass, until the mass change was less than 0.1 mg. The disks were then tested for water sorption (W_sp_) following ISO 4949:2009^40^. The diameter and thickness of each disk were measured individually to calculate the volume (V). Then the disks were immersed in distilled water at 37 °C for 7 d. Then the disk was taken out of the water, its surface was wiped with absorbent paper and the weight was immediately measured using an analytical balance to yield M_WT_. The disk was then placed in the desiccator under vacuum for 7 d until reaching constant weight, yielding M_DRY._ W_sp_ was calculated for each specimen by using the following equation^39^: W_sp_ = (M_WT_ − M_DRY_)/V, expressed in mg/mm^3^.

### Initial Ca and P ion release measurement

A NaCl solution (133 mmol/L) was buffered to pH 4 with 50 mmol/L lactic acid to measure the ion release, simulating a cariogenic low pH condition^18^,^22^. Groups 1–4 were used for Ca and P ions release. Groups 1 and 3 contained no Ca and P ions and therefore served as the negative controls to groups 2 and 4, respectively. Groups 5 and 6 were not tested because they did not contain CaP and therefore had no Ca and P ion release. Three specimens of approximately 2 × 2 × 12 mm were immersed in 50 mL of solution to yield a specimen volume/solution of 2.9 mm^3^/mL, following previous studies^18^,^20^,^22^. This was similar to a specimen volume per solution of about 3.0 mm^3^/mL in a previous study^26^. The concentrations of Ca and P released from the specimens were measured at 1, 3, 5, 7, 14, 21, 28, 35, and 42 d, using methods previously described^18^,^20^,^22^,^41^,^42^. At each time, aliquots of 0.5 mL were removed and replaced by fresh NaCl solution. The pH of the immersion solutions was monitored and adjusted to pH 4 with 50 mmol/L lactic acid using a combination pH electrode (Orion, Cambridge, MA)^23^. The aliquots were analyzed for Ca and P concentrations via a spectrophotometric method (DMS-80 UV-visible, Varian, Palo Alto, CA) using known standards and calibration curves^18^,^20^,^22^. This ion release from the cement specimens was termed the “initial release”, to differentiate from the subsequent recharge and re-release.

### Recharge of cement specimens and Ca and P ion re-release

First, the cement specimens were stored in the pH 4 solution for 42 days to exhaust the ion release^30^,^31^. The specimens were then placed in another fresh pH 4 solution for an additional 30 days to make sure that the ion release was indeed exhausted. These specimens were then subjected to a Ca and P ion release measurement for 7 days to confirm that there was no further release. The exhausted specimens were then used for Ca and P ion recharge. The calcium recharge solution consisted of 100 mmol/L of CaCl_2_ and 50 mmol/L of 4-(2-hydroxyethyl)-1-piperazineethanesulfonic acid (HEPES) buffer^30^,^31^. The phosphate ion recharge solution consisted of 60 mmol/L of KHPO_4_ and 50 mmol/L of HEPES buffer. The two solutions were adjusted to pH 7 using 1 mol/L of KOH^30^,^31^. Three specimens of approximately 2 × 2 × 12 mm were immersed into 5 mL of a recharge solution and gently shaken using a mixing machine (Analog Vortex Mixer, Fisher Scientific, Waltham, MA) for 3 minutes. This was done because there is usually fluid movement and shaking when using a mouthwash. Then the specimens were rinsed with running distilled water for 1 min to remove any loosely attached deposits on specimen surfaces, hence only the ions recharged into the interior of the cement were measured in the subsequent re-release test. The specimens received two doses of recharge, one at about 9:00 am, and the other at about 5:00 pm, which simulated a mouth-rinse in the morning and in the evening. After recharging, the specimens were immersed in 50 mL of pH 4 solution for 7 days to measure the Ca and P ion re-release, using the ion concentration measurement described in the initial Ca and P release measurement. After 7 days of re-release, the specimens were recharged again as described above and tested for re-release as cycle 2. This was repeated for 3 cycles in the present study to examine if the rechargeability and re-release would decrease with increasing the number of cycles.

In order to investigate how long the specimens could further release Ca and P ions after three cycles of recharge/re-release, the specimens after the 3rd recharge (without further recharge) were immersed in 50 mL of fresh pH 4 solution for additional 42 d. The concentrations of Ca and P ions re-released from these specimens were measured at 1, 2, 3, 4, 5, 6, 7, 14, 21, 28, 35 and 42 d as described in the initial Ca and P release measurement^18^,^22^.

### Statistical analysis

Kolmogorov–Smirn test and Levene test were performed to confirm the normality and equal variance of the data. The results of bracket bond strength, water sorption, Ca and P ion release and re-release were analyzed via the analyses of variance (ANOVA). Post hoc multiple comparisons were performed using Tukey’s honestly significant difference test. ARI results were evaluated using the Chi-Square test. Statistical significance was preset at p *<* 0.05. The SPSS 14.0 software package (SPSS, Chicago, IL, USA) was used.

## Results

**Figure 1:**
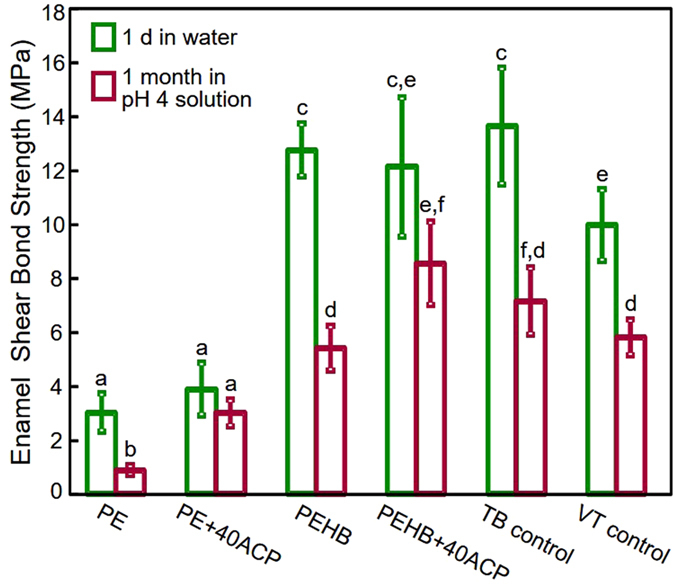
Orthodontic bracket shear bond strength (mean ± sd; n = 10). Green bars represent the specimens tested after 1 day in water. Red bars represent the specimens tested after immersion in demineralization solution of pH 4 for 1 month. Bars with dissimilar letters indicate values that are significantly different from each other (p < 0.05).

The bracket-enamel shear bond strengths are plotted in Fig. 1 (mean ± sd; n = 10). Both cement type and immersion time showed significant effects on the bond strength (p < 0.001). After 1 day in water, PEHB, PEHB+40ACP and TB had similarly high shear bond strengths (12.75 ± 1.00, 12.12 ± 2.60, 13.63 ± 2.18 MPa respectively) (p = 0.728). PE had significantly lower bond strength (p < 0.001). The incorporation of 40% ACP into PEHB had no significant effect on the bond strength, compared to that of PEHB (p = 1). The 1-month immersion in pH 4 solution decreased the shear bond strength of all tested cements (p < 0.001). After 1 month of aging in the demineralization solution, PEHB+40ACP (8.56 ± 1.56 MPa) had a slightly higher shear bond strength than TB (7.15 ± 1.26 MPa) (p = 0.14), but a significantly higher bond strength than PEHB (5.40 ± 0.83 MPa) and VT control (5.80 ± 0.67 MPa) (p < 0.001). Although there was a reduction in the shear bond strength of the 1 month aged specimens for the PEHB+40ACP group, there was no significant difference with the specimen group after 1 day in water.

The bracket-enamel shear bond ARI results are listed in Table 1. There were significant effects of cement type and aging time (p_cement_ = 0.03; P_time_ = 0.001). After 1 day in water, more cements remained on the enamel surfaces in PEHB group than PE (p = 0.018). There was no significant difference in ARI between PEHB, PEHB+40ACP, TB control and VT control (p = 0.881). After 1 month immersion in demineralization solution at pH 4, the ARI scores significantly decreased (all p < 0.05). The ARI scores in PEHB+40ACP and TB control were significantly higher than those in other groups (all p < 0.05).

**Figure 2:**
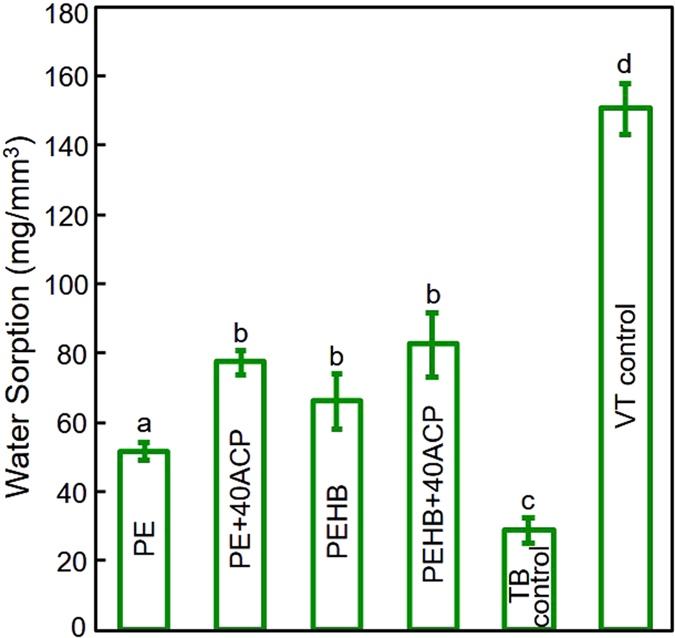
Results for water sorption (mean ± sd; n = 3). Bars with dissimilar letters indicate values that are significantly different from each other (p < 0.05).

The W_SP_ results of the cements are plotted in Fig. 2 (mean ± sd; n = 3). VT control had the highest W_SP_ while TB had the lowest (p < 0.001). The incorporation of 40% ACP into PEHB did not significantly increase the W_SP_ in relation to PEHB. (p = 0.727). PEHB and PEHB+40ACP both had intermediate W_SP_ values that were in between the two commercial materials.

**Figure 3:**
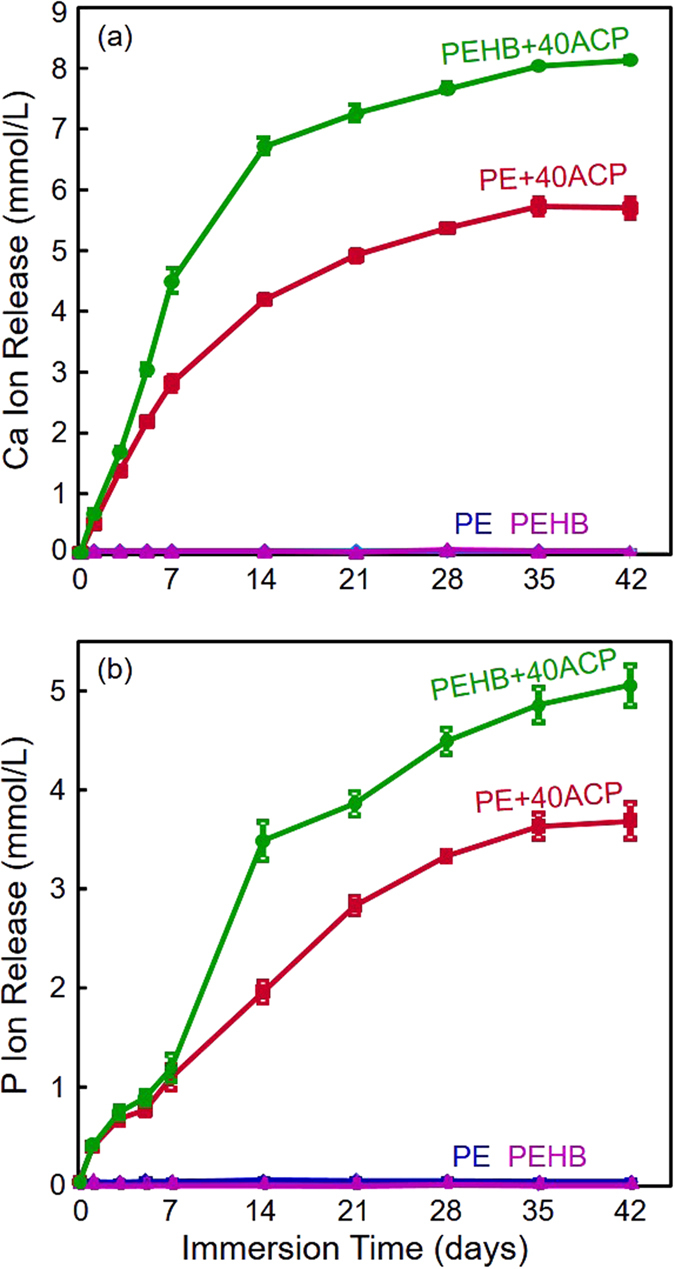
Initial Ca and P ion release (mean ± sd; n = 6) from the orthodontic cements: (**a**) Ca ion release, and (**b**) P ion release. PEHB+40ACP showed significantly higher Ca and P ion releases than PE+40ACP (p < 0.05).

The initial Ca and P ion releases from the cements are plotted in Fig. 3 (mean ± sd; n = 6). PE + 40ACP and PEHB+40ACP had high levels of ion release. Ion concentrations increased with time (all p < 0.05). Both Ca and P ion releases from PEHB+40ACP were significantly greater than those from PE + 40ACP (for calcium, p < 0.001; for phosphate, p < 0.001), indicating that the ion release was significantly affected by the resin compositions.

**Figure 4:**
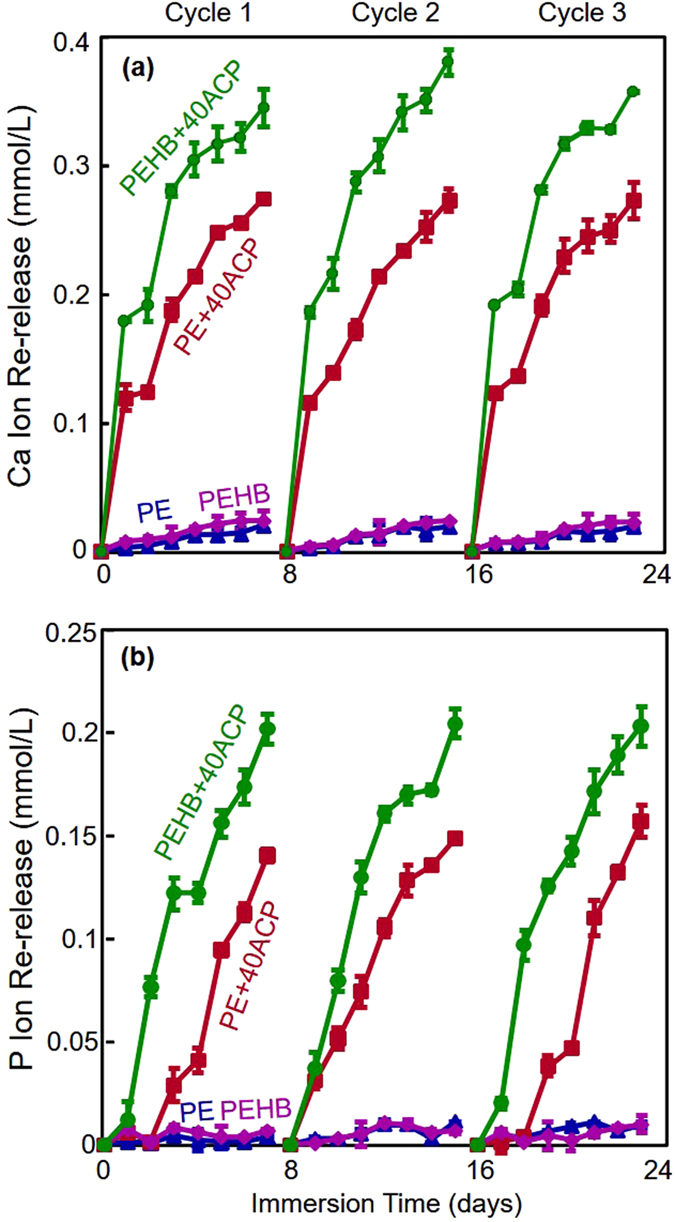
Ca and P ion recharge and re-release (mean ± sd; n = 3). (**a**) Ca ion re-release. (**b**) P ion re-release. Ca and P ion re-releases of PEHB+40ACP were significantly higher than those of PE+40ACP (p < 0.001). There was no decrease in the ion re-release level with increasing the number of recharge/re-release cycles from cycle 1 to cycle 3 (p > 0.1).

The Ca and P ion recharge and re-release results are plotted in Fig. 4 (mean ± sd, n = 3). Three recharge/re-release cycles were included, with each recharge being tested for re-release for 7 days. Cements with 0% ACP (PE and PEHB) showed little Ca and P ion re-release after being immersed in the recharge solution. In contrast, PE+40ACP and PEHB+40ACP (which were exhausted in ion release prior to the recharge) showed high levels of Ca and P ion re-release after each recharge. The ion concentrations increased rapidly from 1 to 7 days. Furthermore, there was no decrease in ion re-release from the first recharge/re-release cycle to the third cycle. For each cycle, the ion release reached a similarly high level. These results demonstrate the potential for a long-term ion recharge/re-release capability. In addition, PEHB+40ACP showed significantly greater recharge/re-release of ion concentrations than PE+40ACP (Cycle 1, calcium, p < 0.001; phosphate, p = 0.047. Cycle 2, calcium, p = 0.004; phosphate, p < 0.001. Cycle 3, calcium, p = 0.012; phosphate, p < 0.001).

**Figure 5:**
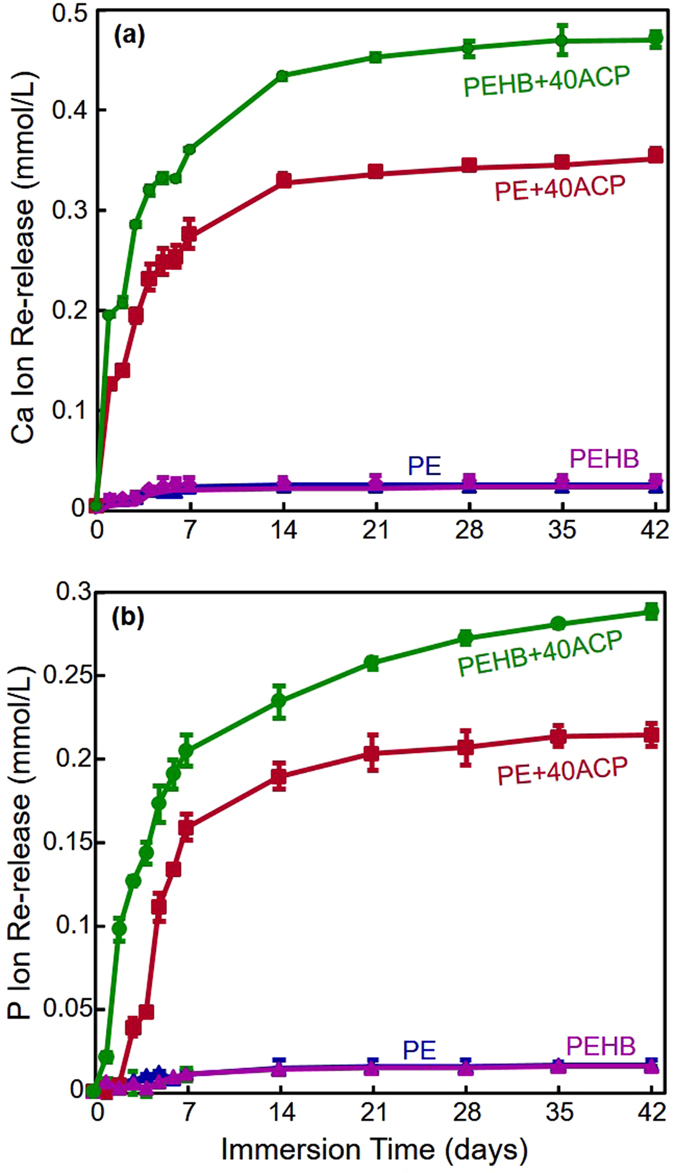
Continuous Ca and P ion re-release after the third recharge (mean ± sd; n = 3), without further recharge. PEHB+40ACP had higher Ca and P re-release than PE+40ACP (p < 0.01). The ion concentration increased for about 21 days and then gradually plateaued, indicating that after the third recharge cycle and releasing ions for 7 days in Fig. 4, the cements continuously released ions for three more weeks without further recharge.

After the third recharge/re-release cycle, the specimens were tested for Ca and P ion release for 42 days without any further recharge. The results are plotted in Fig. 5 (mean ± sd, n = 3). The released ion concentrations increased form 1 day to about 21 days and then gradually reached a plateau. These results demonstrate that, after a recharge and after releasing ions continuously for 7 days (in Fig. 4), the cements without any recharge could release Ca and P ions for another 21 days continuously. These results demonstrate that it is not necessary to recharge daily or weekly; one recharge of the orthodontic cement could yield about four weeks of ion release. In addition, PEHB+40ACP exhibited significantly higher Ca and P ion re-release than PE+40ACP (calcium, p = 0.005; phosphate, p < 0.001).

## Discussion

Orthodontic patients are often at a high risk of developing dental caries during orthodontic treatment, especially when their compliance to oral hygiene instructions is doubtful^43^,^44^. In addition, in fixed orthodontic treatments, the enamel surface is often etched with phosphoric acid, which produces the appropriate etching pattern to facilitate bracket bonding. However, the entire etched enamel surface usually cannot be completely covered by orthodontic cement because it is clinically impossible to visualize the small area of etched enamel. The etched enamel surface, not covered by the orthodontic cement is vulnerable to the retention of oral micro-organisms and the resultant demineralization that results in WSL and caries^45^. Mature enamel is an acellular matrix, thus it is impossible to regenerate after trauma or decay^46^. Therefore, the repair of vulnerable enamel can only be accomplished by using extraneous materials. CaP orthodontic cements that are capable of releasing high level of Ca and P ions would allow freely available Ca and P ions to enter enamel to prevent demineralization and enhance reminralization^15^,^47^. In the present study, a novel 40% ACP orthodontic cement with capability of repeated Ca and P ion recharge and re-release was developed. Among the four tested ACP cements, cement PEHB+40ACP showed the highest Ca and P ion release and recharge capability, as well as a high bracket-enamel bond strength that had a good resistance to acid attacks. The hypotheses were proven that the new rechargeable CaP cement had a bracket-enamel bond strength similar to commercial orthodontic cements without CaP; that the enamel bond strength and the Ca and P ion recharge and re-release depended on cement composition; and that the new CaP cement produced durable ion release which did not decrease with increasing the number of recharge and re-release cycles.

The novel rechargeable PEHB+40ACP cement could be especially advantageous in the reduction of WSL because it has the potential to be repeatedly recharged with Ca and P ions using easy and user-friendly recharging protocols such as a mouth-rinse. Indeed, the results of the present study indicate that: (1) one recharge treatment has the potential to provide weeks of ion release; (2) the recharge efficacy and re-release did not decrease with increasing number of recharge cycles. Therefore, the PEHB+40ACP orthodontic cement is promising to provide the much needed long-term caries-inhibition and enamel remineralization capability to orthodontic treatments.

For fix orthodontic treatment, a strong bond between bracket and enamel surface is important to provide enough support for bands and wires during the orthodontic process. The present study showed that the resin matrix of orthodontic cements had a significant effect on the bracket bond strength. All the experimental cements in the present study contained PMGDM and EBPADMA. PMGDM is an acidic adhesive monomer that can chemically chelate with calcium or phosphate ion from dentin or from the exterior environment^32^,^48^. PEHB showed significantly higher bracket bond strength than PE. PEHB contained PMGDM and EBPADMA, plus 10% HEMA and 5% BisGMA. HEMA has long been used in dental adhesives^31^. It can improve the hydrophilicity and flowability of the cement and facilitate a closer contact with the demineralizaed tooth structure^33^,^49^, producing cross-linked interlocks between the adhesive and tooth hard tissues. BisGMA is frequently used in adhesives and composites^33^,^50^. BisGMA contains ester linkages that connect Bis-phenol-A segments to the polymerizable vinyl segments. Due to its high molecular weight, BisGMA provides a lower polymerization shrinkage and rapid hardening and the polymer had good mechanical properties^33^,^50^. These factors contributed to the relatively high bracket bond strength of about 13 MPa for PEHB, which was much higher than that of PE and approached that of TB control. The use of BisGMA-rich and HEMA-rich bonding agents has raised concerns of adhesive hydrolysis and degradation of the bonded interfaces^51^,^52^. Some advanced cements such as Vitremer have been reported to release HEMA after setting, which might increase the risks of adverse pulpal responses in patients and the allergy in patients and dental personnel^53^. Therefore, in the present study, PEHB used relatively small amounts of 10% HEMA and 5% BisGMA. In addition, while dentin is an organic and water rich bonding substrate, enamel contains 95–97% of apatite crystals. The bonding mechanism of enamel is mechanical interlock and chemical chelation between demineralized enamel and the adhesive monomers^54^. The bonding is based on the enamel matrix being typically dried to a chalky color and thus less susceptible to water. Therefore, most bonding agents containing HEMA and BisGMA illustrated strong and durable bonding to enamel in both laboratory studies and in the clinical setting^55^.

The incorporation of 40% ACP into the PE and PEHB, while providing beneficial ion release and recharge, did not have a negative effect on the bracket bond strength when PE and PEHB was compared to PE+40ACP and PEHB+40ACP. After 1 month of aging in a demineralization solution, the bracket bond strength of most of the tested cements decreased. The demineralization solution of pH 4 was used to mimic demineralizing oral fluid conditions and biofilm acids that may cause the degradation of enamel and the bonded interface, which may contribute to decreases in the bond strength. However, PE+40ACP and PEHB+40ACP showed no significant decrease in bond strength after 1 month of aging in the demineralization solution. In particular, PEHB+40ACP showed the highest bracket-enamel bond strength after 1 month of aging. This was likely because PE+40ACP and PEHB+40ACP could release high levels of Ca and P ions which could neutralize acids and raise the acidic pH, thus reducing the damaging effect of acid to enamel and to the bonded interface^23^. In addition, the released ions may help repair the micro-gaps and micro-voids along the enamel-resin interfaces, remineralize and protect the enamel at the interface, thus yielding a strong and durable bracket-enamel bond. Further study can be recommended to investigate the effects of PEHB+40ACP and its recharge on long-term bond strength and enamel protection during acid attacks for longer than a month, such as for one year.

Traditional CaP-containing dental resins used particles with sizes of about 1–55 μm^25^,^26^,^27^. The ACP in the present study had much smaller sizes with a mean particle size of 116 nm^19^. ACP had a high surface area of 17.76 m^2^/g, compared to 0.5 m^2^/g of traditional particles in previous studies^19^,^25^,^26^,^27^. This would increase the amount of interfaces between ACP and the resin matrix, producing more pathways for the diffusion of water and ions^18^. With the same ACP content, PEHB+40ACP showed a significantly higher Ca and P ion release than PE+40ACP. The smaller particle size of 116nm increases the surface area per percentage of particles that would have contact with the tooth structure and the oral environment. With the incorporation of HEMA, the water sorption in PEHB was also increased over that of PE (Fig. 2). Previous studies also showed increasing the HEMA content resulted in an increase in water sorption^49^,^56^,^57^,^58^,^59^. This may promote the water diffusion into the resin and the ion release as well as recharge and long-term re-release. The ion release may produce a supersaturated calcium phosphate environment that can not only inhibit demineralization, but also remineralize the enamel.

The recharge mechanism of the CAP cement appeared to consist of two factors. The first likely was the space-occupying effect. After the initial Ca and P ion release, the sites that were previously occupied by the Ca and P ions would be available for the incoming Ca and P ions from the recharge solution. This may explain why PE and PEHB containing 0% ACP showed little recharge and re-release, as there was no available space in the cement to accept incoming ions. According to this space-occupying mechanism, the material with the higher initial Ca and P ion release should also have a higher Ca and P recharge and re-release. The results of the present study confirmed this hypothesis: Cement PEHB+40ACP with higher initial ion release also showed significantly higher re-release than PE+40ACP. Second, the chemical properties of the cement matrix likely also contributed to the recharge mechanism. Our previous study showed that, with the same content of ACP fillers, the resin of PMGDM-EMBPDMA at 1:1 ratio had a significantly higher Ca and P recharge ability than that of the BisGMA-based and Bis[2-(methacryloyloxy)ethyl] phosphate (BisMEP)-based resins^30^. Therefore, the present study selected PMGDM and EMBPDMA as the major monomers in orthodontic cement. The carboxylate groups of PMGDM can chelate with Ca ions in dentin and from the exterior environment^32^,^48^, such as from the recharge solution. The chelation of calcium ions into the resin and the re-release of calcium from the resin should be a process that depends on the local pH. The recharge solution was at pH 7 to simulate a neutral pH mouth-rinse. The solution for release and re-release testing was at pH 4, to simulate the needed release of these ions triggered by an acid challenge. PMGDM in the cement may chelate with the free calcium in the recharge solution of pH 7 during the recharge. After the recharge and in the pH 4 acid challenge, the bound between PMGDM and calcium might break down to produce the re-release of calcium ions. Further study is needed to investigate and confirm the Ca and P ion recharge and re-release mechanisms and to further improve the recharge efficacy.

Among orthodontic patients, teenagers are the major group. These patients in general have more dynamic and abrasive oral environments due to their diet habits, as well as relatively poor compliance in oral hygiene control. These factors place the young orthodontic patients under higher risks of WSL. The results of the present study indicate that the new orthodontic cement could be recharged once in the morning and once in the evening for a total of two doses of recharge and then it could release Ca and P ions for four weeks without further recharge. Therefore, it may be clinically possible to use the Ca and P recharge solution for mouth-rinse for one day to have lasting release for four weeks, which would be user friendly, especially for teenagers. Alternatively, the recharge of Ca and P ions can be performed in the clinic by dentists at monthly orthodontic appointments. Further studies are needed to optimize the recharge method and determine the clinical efficacy.

Based on the shear bond strength and the Ca and P release for the application as an orthodontic cement, future research with the PEHB+40ACP can include comparative investigations for extended applications to pit and fissure sealants. The use of the PEHB+40ACP with HEMA would need to be assessed for the cytotoxicity of the material before it is used on exposed dentinal root caries and as permanent or temporary restorations.

## Conclusion

Novel dental cement capable of Ca and P ion recharge and long-term release was developed to inhibit tooth demineralization and promote remineralization. Cement PEHB+40ACP showed a relatively high orthodontic bracket-enamel bond strength which was more resistant to acid challenge than that without ACP. PEHB+40ACP had higher initial Ca and P ion release and greater capability for Ca and P ion recharge and re-release, than PE+40ACP. After one recharge, the cement had continuous release of ions for about four weeks, before another recharge would be needed. There was no decrease in recharge and re-release efficacy with increasing number of recharge/re-release cycles.

## Additional Information

**How to cite this article**: Zhang, L. *et al*. Rechargeable calcium phosphate orthodontic cement with sustained ion release and re-release. *Sci. Rep.* **6**, 36476; doi: 10.1038/srep36476 (2016).

**Publisher’s note:** Springer Nature remains neutral with regard to jurisdictional claims in published maps and institutional affiliations.

## Figures and Tables

**Table 1:** Adhesive remnant index (ARI) after orthodontic bracket shear bond testing.

Cements	Aging treatments	ARI Scores				SS
		0	1	2	3	
PE	1 day in water	7	3	0	0	a
PE+40ACP	1 day in water	8	2	0	0	a
PEHB	1 day in water	2	3	5	0	c
PEHB+40ACP	1 day in water	2	4	4	0	c
TB control	1 day in water	2	4	4	0	c
VT control	1 day in water	2	5	3	0	c
PE	1 month in pH 4 solution	9	1	0	0	a
PE+40ACP	1 month in pH 4 solution	9	1	0	0	a
PEHB	1 month in pH 4 solution	7	2	1	0	a
PEHB+40ACP	1 month in pH 4 solution	4	4	2	0	b
TB control	1 month in pH 4 solution	5	4	1	0	b
VT control	1 month in pH 4 solution	7	3	0	0	a

Each group has n = 10. SS refers to statistical significance, with different letters indicating significant differences in the ARI scores (p < 0.05).
